# Supplementary material for: Genome-wide CRISPR/Cas9 deletion screen defines mitochondrial gene essentiality and identifies routes for tumour cell viability in hypoxia
Source: Commun Biol. 2021 May 21;4:615. doi: 10.1038/s42003-021-02098-x (PMC8140129; doi:10.1038/s42003-021-02098-x)
Supplement: Supplementary file 2 — Supplementary Information [file 42003_2021_2098_MOESM2_ESM.pdf]

Supplementary Information. Thomas et al.

Genome-wide CRISPR/Cas9 deletion screen defines mitochondrial gene essentiality and identifies routes for tumour cell viability in hypoxia.

Luke W. Thomas, Cinzia Esposito, Rachel E. Morgan, Stacey Price, Jamie Young, Steven P. Williams, Lucas A. Maddalena, Ultan McDermott and Margaret Ashcroft.

Description of Supplementary Information.

Supplementary Methods.

Gene expression analysis; Mitochondrial copy number.

Supplementary Figures.

Supplementary Fig. 1: Genome-wide CRISPR deletion screen in normoxia, hypoxia, and glucose-free galactose conditions.

Supplementary Fig. 2: Genome-wide CRISPR deletion screen in normoxia, hypoxia, and glucose-free galactose conditions

Supplementary Fig. 3: CRISPR deletion screen identifies enriched genes in normoxia-glucose conditions.

Supplementary Fig. 4: Hypoxia promotes loss of mitochondrial genes and genes involved in energy-consuming processes.

Supplementary Fig. 5: Hypoxia promotes loss of mitochondrial genes and genes involved in energy-consuming processes.

Supplementary Fig. 6: Loss of SDHC improves tumour cell growth in hypoxia.

Supplementary Fig. 7: Galactose sensitises tumour cells to loss of mitochondrial genes, and promotes loss of genes involved in energy-consuming processes.

Supplementary Fig. 8: Inhibitory effects of rotenone in normoxia and hypoxia.

Supplementary Fig. 9: Unprocessed western blots and ponceau S-stained membranes.

Supplementary Fig. 10: Unprocessed western blots and ponceau S-stained membranes.

Supplementary Tables.

Supplementary Table 1: Selected enriched mitochondrial sgRNAs for hypoxia vs normoxia.

Supplementary Table 2: QPCR primer sequences.

Supplementary Table 3: siRNA sequences.

Supplementary Information. Thomas et al.

Supplementary Methods.

Gene expression analysis. Related to Fig. 3 and Supplementary Fig. 6.

Total RNA samples were isolated using the GeneElute kit, following the manufacturer's protocol (Sigma-Aldrich). cDNA synthesis was carried out using the qScript synthesis kit, following the manufacturer's protocol (Quantabio). mRNA expression was measured by quantitative (Q)-PCR using SYBR Green Mastermix (Eurogentec Ltd.) and the DNA Engine Opticon 2 system (BioRad). Q-PCR primer sequences are outlined in Supplementary information Table 1.

Mitochondrial copy number. Related to Supplementary Fig. 5.

Measurement of mitochondrial (mt)DNA copy number has been described by us previously [9]. Total DNA samples were isolated using the QIAamp DNA Blood Mini Kit, following the manufacturer's protocol (Sigma-Aldrich). Relative copy numbers of the single-copy nuclear-encoded gene beta-2-microglobulin (*B2M*) and the mitochondrially encoded gene *MTND1* were measured by quantitative (Q)-PCR using SYBR Green Mastermix (Eurogentec Ltd.) and the DNA Engine Opticon 2 system (BioRad). The Q-PCR primer sequences are in Supplementary Table 1. To determine the mtDNA content, relative to nuclear DNA, the following equations were used:

a. $\Delta CT = (\text{nucDNA CT} - \text{mtDNA CT})$
b. $\text{Relative mtDNA content} = 2 \times 2\Delta CT$

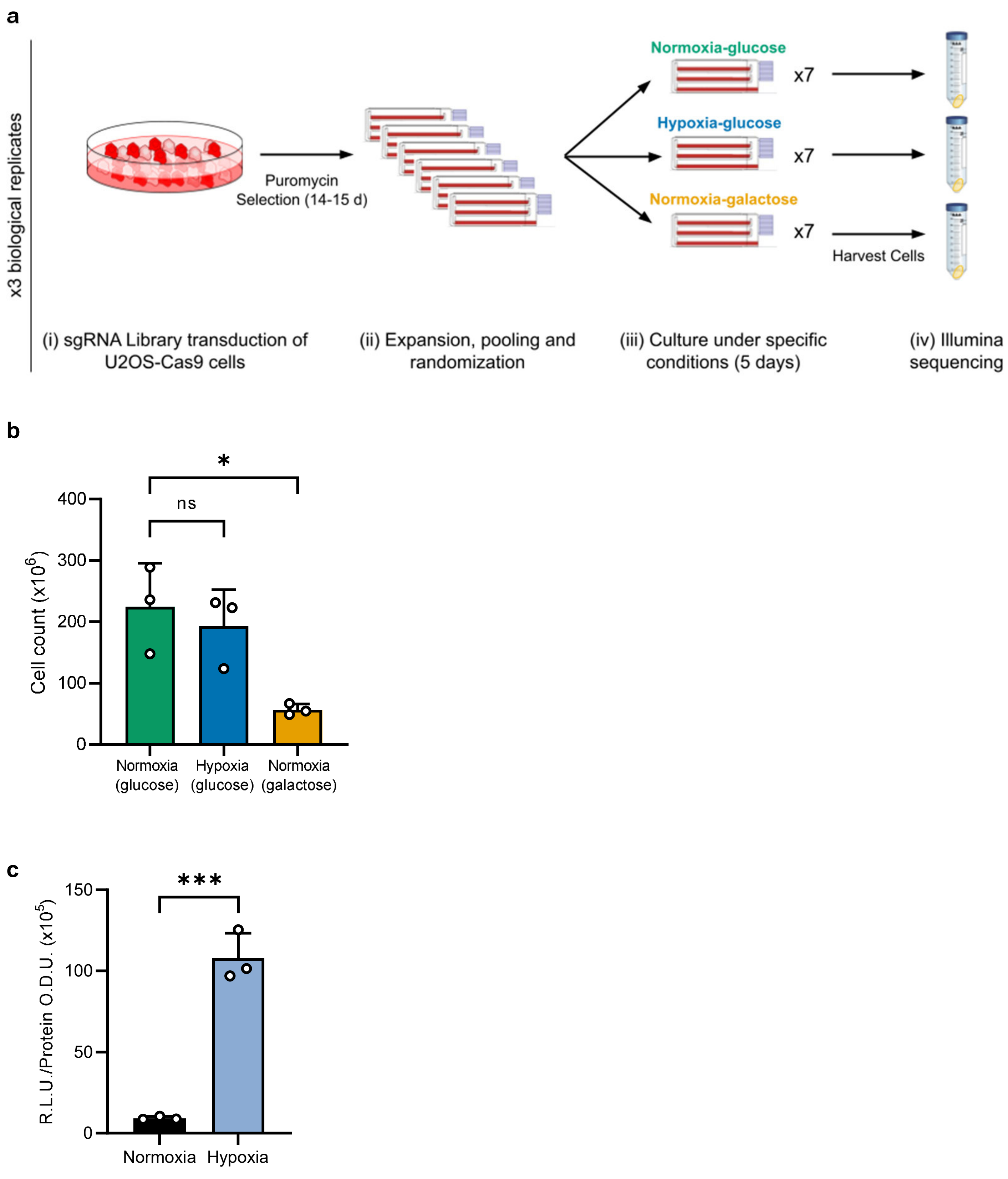

Supplementary Fig. 1: Genome-wide CRISPR deletion screen in normoxia, hypoxia, and glucose-free galactose conditions.

Supplementary Fig. 1: Genome-wide CRISPR deletion screen in normoxia, hypoxia, and glucose-free galactose conditions. a, Schematic shows experimental design of genome-wide CRISPR/Cas9 deletion screen. U2OS-Cas9 cells were transduced with a lentiviral sgRNA library targeting 18,009 genes (i), and transduced cells were selected by puromycin for 14-15 days (ii). Cells were then cultured in 25 mM glucose in either normoxia (normoxia-glucose) or hypoxia (1% O_2) (hypoxia-glucose), or in 25 mM galactose in normoxia (normoxia-galactose) for 5 days (iii), prior to harvesting and sequencing for sgRNA abundance (iv). Sequencing reads from triplicate incubations were analysed by the MAGeCK analysis platform. Library versus plasmid comparisons were performed for each condition, and relative sgRNA abundances were calculated between experimental conditions. Three independent biological repeat screens (i-iv) were performed. **b,** Chart shows total cell counts of the U2OS-Cas9 library cells cultured for 5 days for each condition from our screen described in (**a**). n=3; mean±S.D.; n.s. not significant; *p<0.05. **c,** Chart shows HRE luciferase activity (RLU) relative to total cell protein in U2OS-HRE cells cultured in glucose and incubated in normoxia or hypoxia (1% O_2) for 5 days. n=3 technical replicates; mean±S.D; **p<0.01.

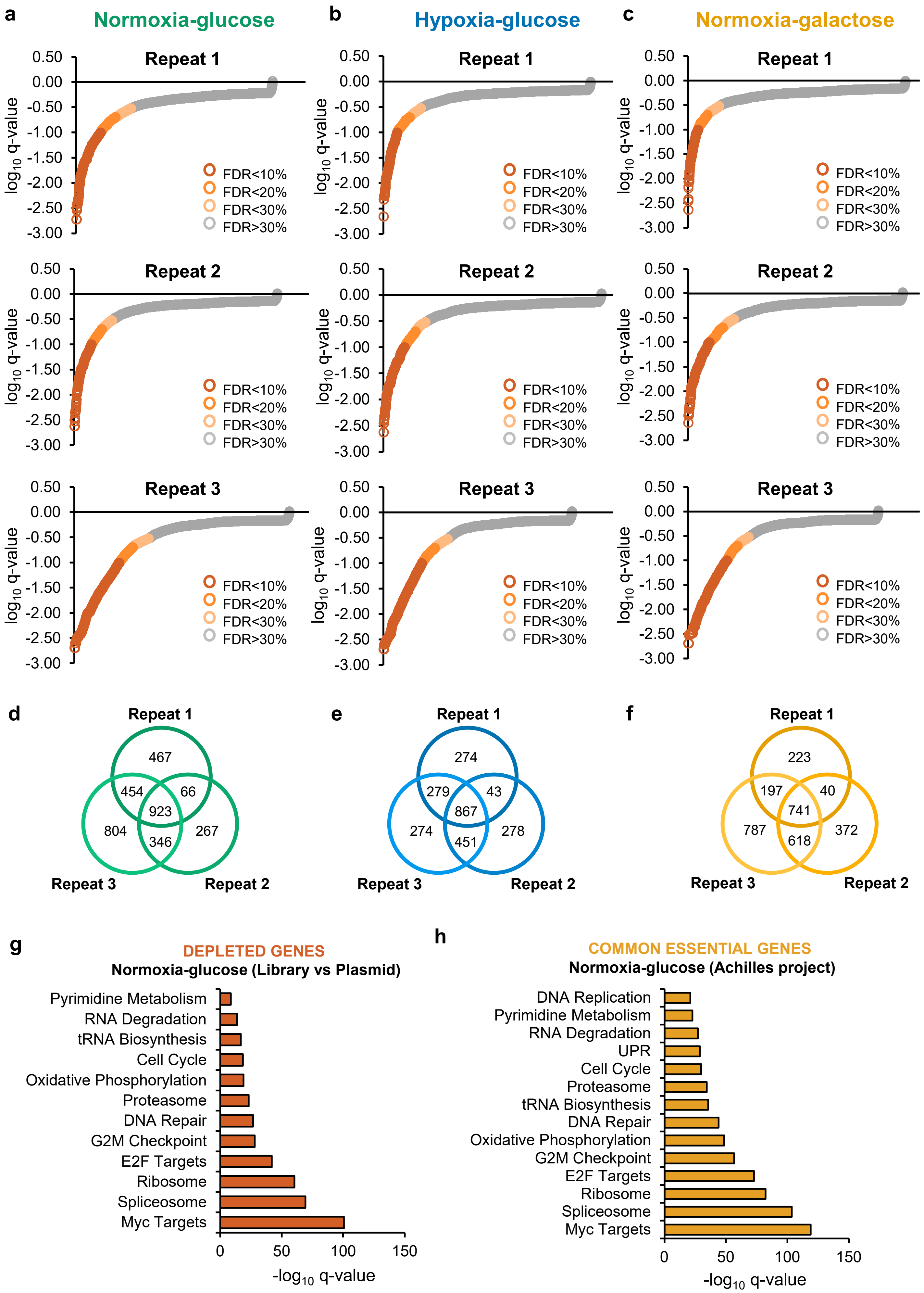

Supplementary Fig. 2: Genome-wide CRISPR deletion screen in normoxia, hypoxia, and glucose-free galactose conditions.

Supplementary Fig. 2: Genome-wide CRISPR deletion screen in normoxia, hypoxia, and glucose-free galactose conditions. a-c, Charts show FDR-corrected significance values of all genes with significantly depleted sgRNAs at different FDR thresholds in U2OS-Cas9 library cells compared to plasmid control, and cultured under the indicated conditions from the screen described in Supplementary Fig 1a**.** Each of the three biological replicates are shown independently for each condition (normoxia-glucose; hypoxia-glucose; normoxia-galactose). **d-f**, Venn diagrams show proportion of overlapping genes with significantly depleted sgRNAs from each replicate in (**a-c**). **g,** Chart shows overrepresentation analysis of all genes with significantly depleted sgRNAs from all replicates in (**a**). **h,** Chart shows overrepresentation analysis of the common essential gene list from the Achilles project, cancer dependency map (DepMap).

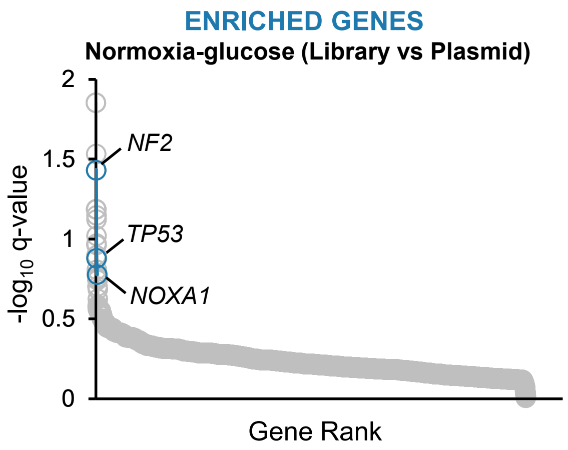

Supplementary Fig. 3: CRISPR deletion screen identifies enriched genes in normoxia-glucose conditions. Chart shows FDR-corrected significance values of all genes with significantly enriched sgRNAs from U2OS-Cas9 cells transduced with the genome-wide sgRNA library compared to plasmid control for 5 days in normoxia-glucose conditions. Selected genes are highlighted in blue. n=3.

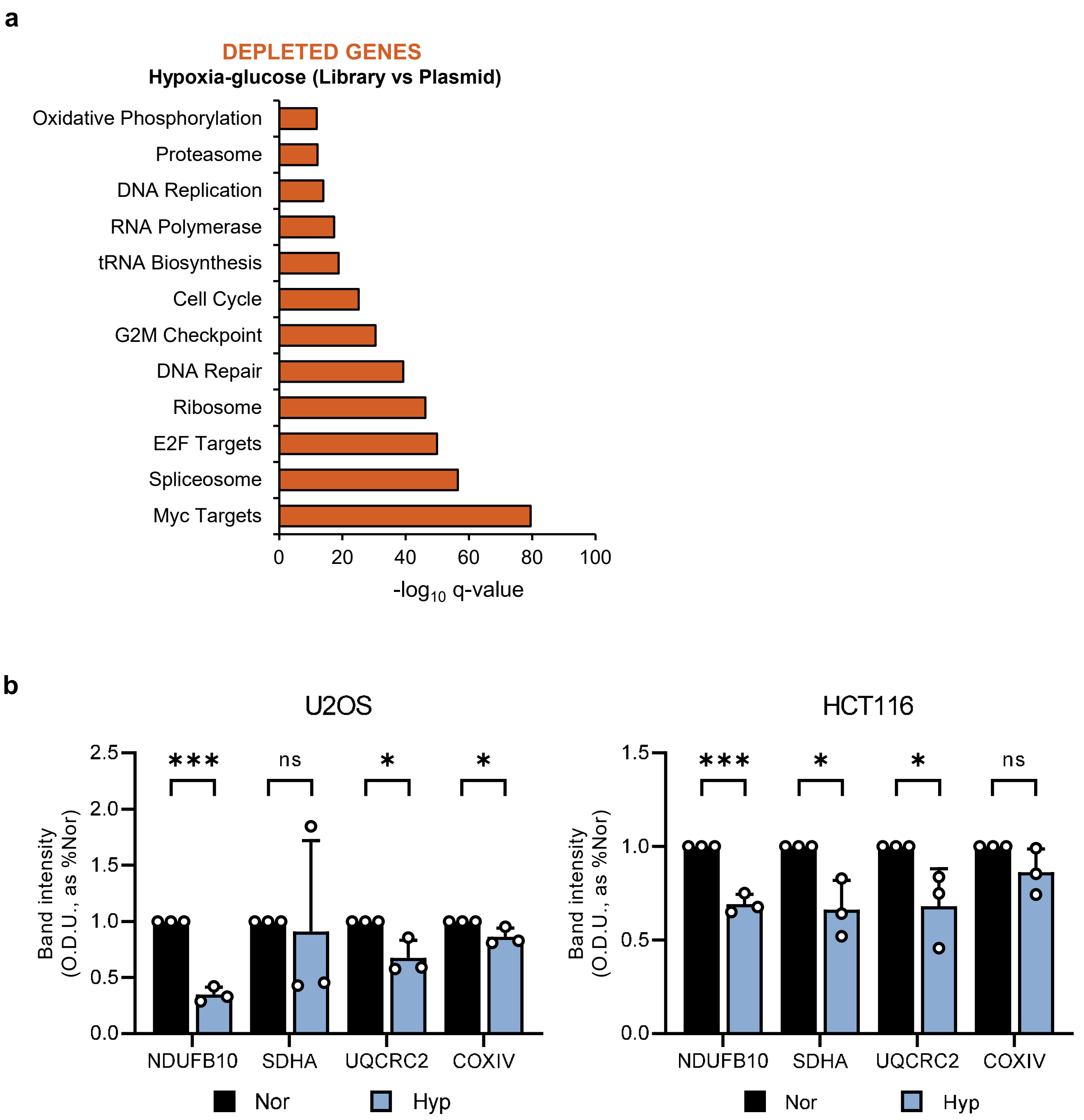

Supplementary Fig. 4: Hypoxia promotes loss of mitochondrial genes and genes involved in energy-consuming processes. a, Chart shows overrepresentation analysis of all genes with significantly depleted sgRNAs from cells incubated in hypoxia-glucose, transduced with the sgRNA library compared to the plasmid controls. n=3. **b,** Charts show relative density of protein bands from western blots in Fig. 2e. Each protein band density value was normalised to the respective β-Actin (load control) density value. Data represented as a % relative to normoxic conditions. n=3; mean±S.D.; n.s. not significant; *p<0.05; ***p<0.001.

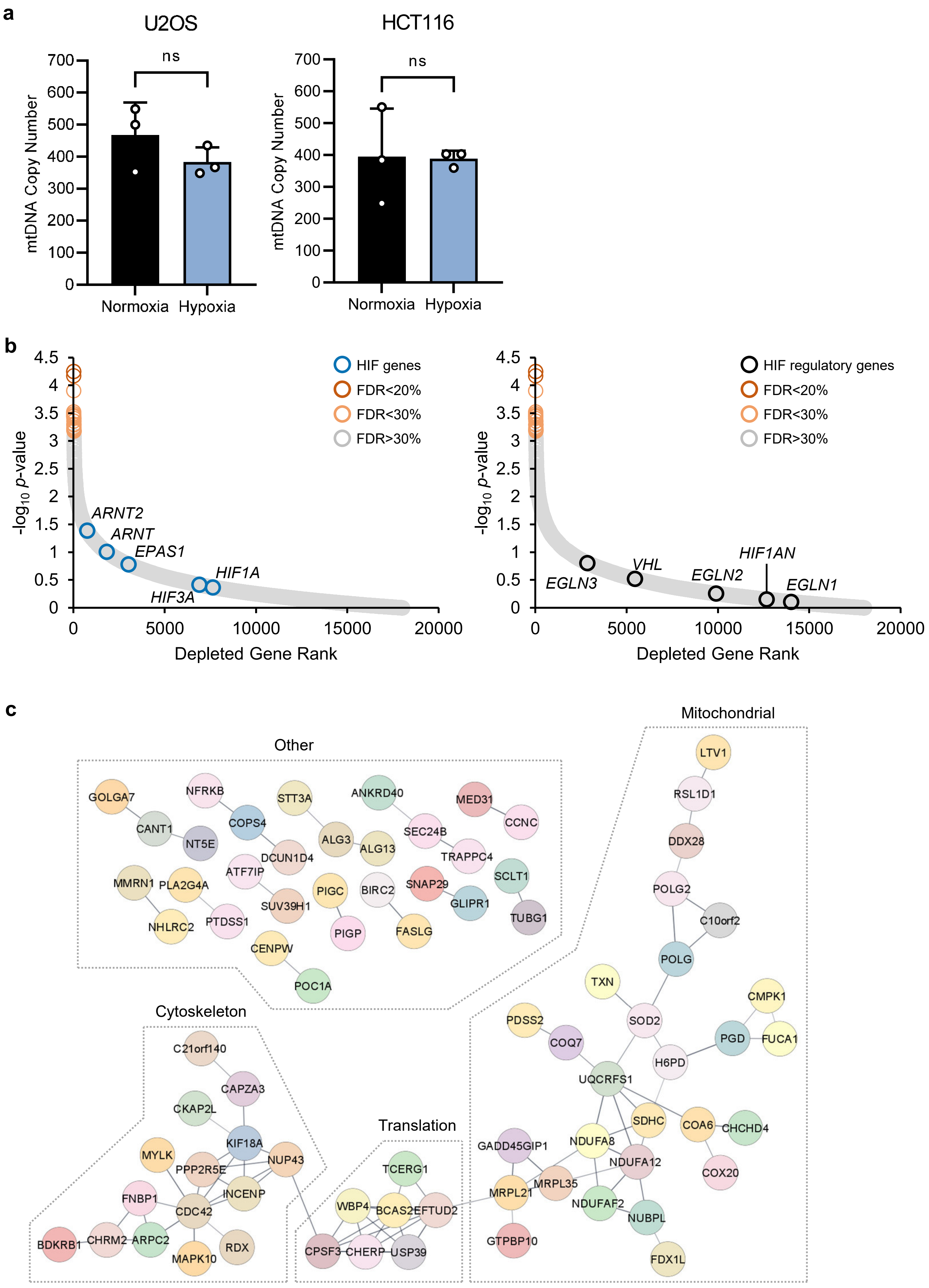

Supplementary Fig. 5: Hypoxia promotes loss of mitochondrial genes and genes involved in energy-consuming processes.

Supplementary Fig. 5: Hypoxia promotes loss of mitochondrial genes and genes involved in energy-consuming processes. a, Charts show mtDNA copy number calculated by ratio of *MT-ND1* and *B2M* gene abundance in U2OS and HCT116 cells incubated for 5 days in either normoxia-glucose or hypoxia-glucose (1% O_2). n=3; mean±S.D.; n.s. not significant. **b,** Charts show significance values (-$\log_{10}$ p-value) of all significantly depleted genes in hypoxia-glucose compared to normoxia-glucose at different FDR thresholds (<20%, <30%, >30%). HIF family genes (left chart), and HIF pathway regulatory genes (right chart) are highlighted in blue and black respectively. **c**, Schematic diagram shows interacting genes from all genes with significantly enriched sgRNAs in hypoxia-glucose compared to normoxia-glucose from our screen described in Supplementary Fig. 1a. Nodes of genes with similar functions are boxed, as indicated.

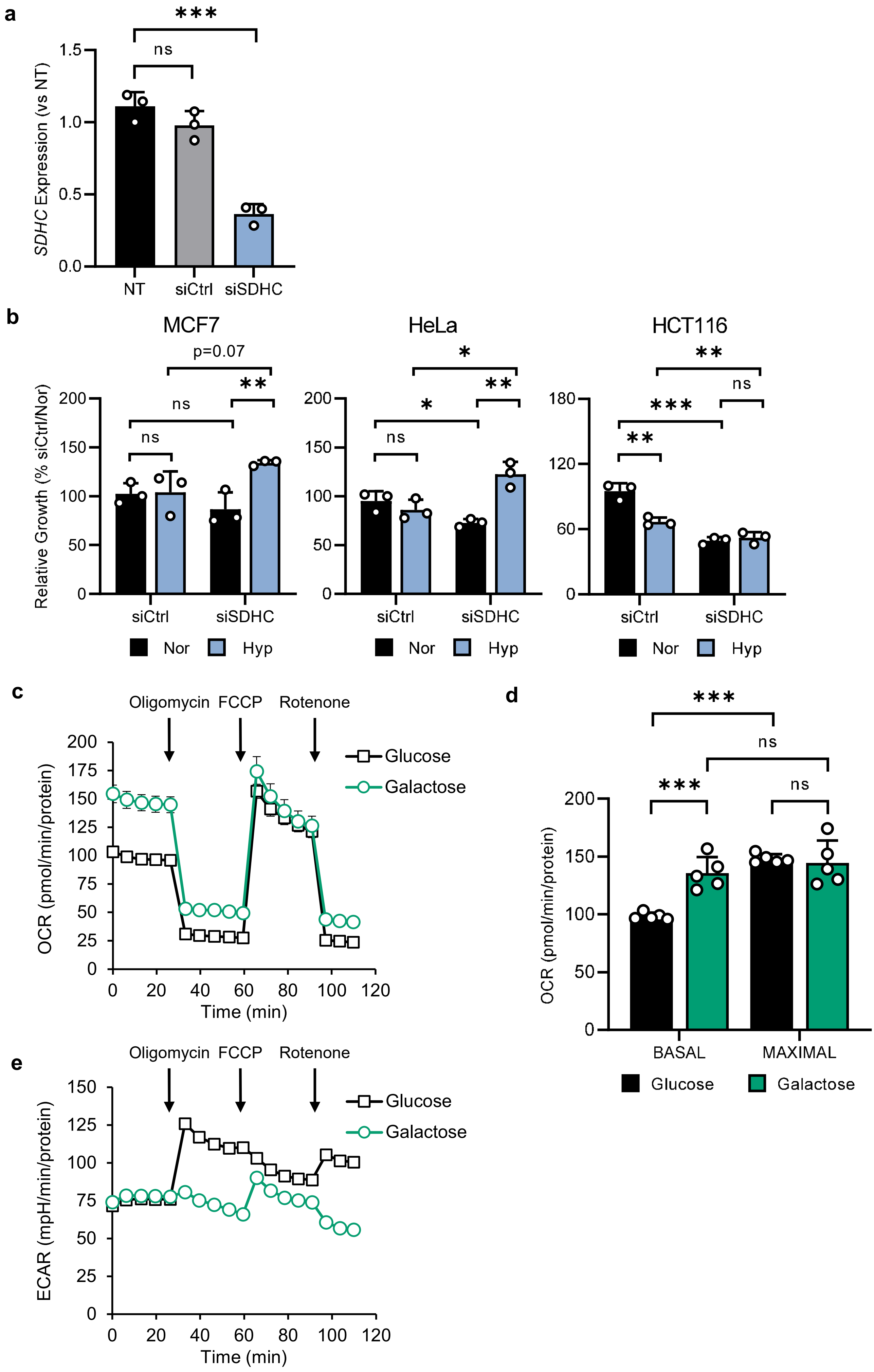

Supplementary Fig. 6: Loss of SDHC improves tumour cell growth in hypoxia.

Supplementary Fig. 6: Loss of SDHC improves tumour cell growth in hypoxia. a, Chart shows *SDHC* expression in U2OS cells treated with non-targeting control siRNA (siCtrl), siRNA targeting *SDHC* (siSDHC) or untreated cells (NT) for 5 days in normoxia-glucose. n=3; mean±S.D.; n.s. not significant; ***p<0.001. **b,** Charts show relative % growth of HCT116, HeLa, and MCF-7 cells cultured for 5 days in normoxia-glucose or hypoxia-glucose (1% O_2), treated with non-targeting control siRNA (siCtrl) or siRNA targeting *SDHC* (siSDHC). n=3. mean±S.D.; n.s. not significant; *p<0.05; **p<0.01; ***p<0.001. **c**, Chart shows oxygen consumption rate (OCR) for U2OS-HRE cells cultured in 25 mM glucose or galactose, treated serially with oligomycin, FCCP, and rotenone. Data represented as pmol/min relative to total cellular protein measured (pmol/min/protein). mean±S.E.M. n=48 technical replicates. **d**, Chart shows basal and maximal OCR of cells in (**c**). Data represented as pmol/min relative to total cellular protein measured (pmol/min/protein). n=5; mean±S.D.; n.s. not significant; ***p<0.001. **e**, Chart shows extracellular acidification rate (ECAR) of cells in (**c**). Data represented as mpH/min relative to total cellular protein measured (mpH/min/protein). mean±S.E.M. n=48 technical replicates.

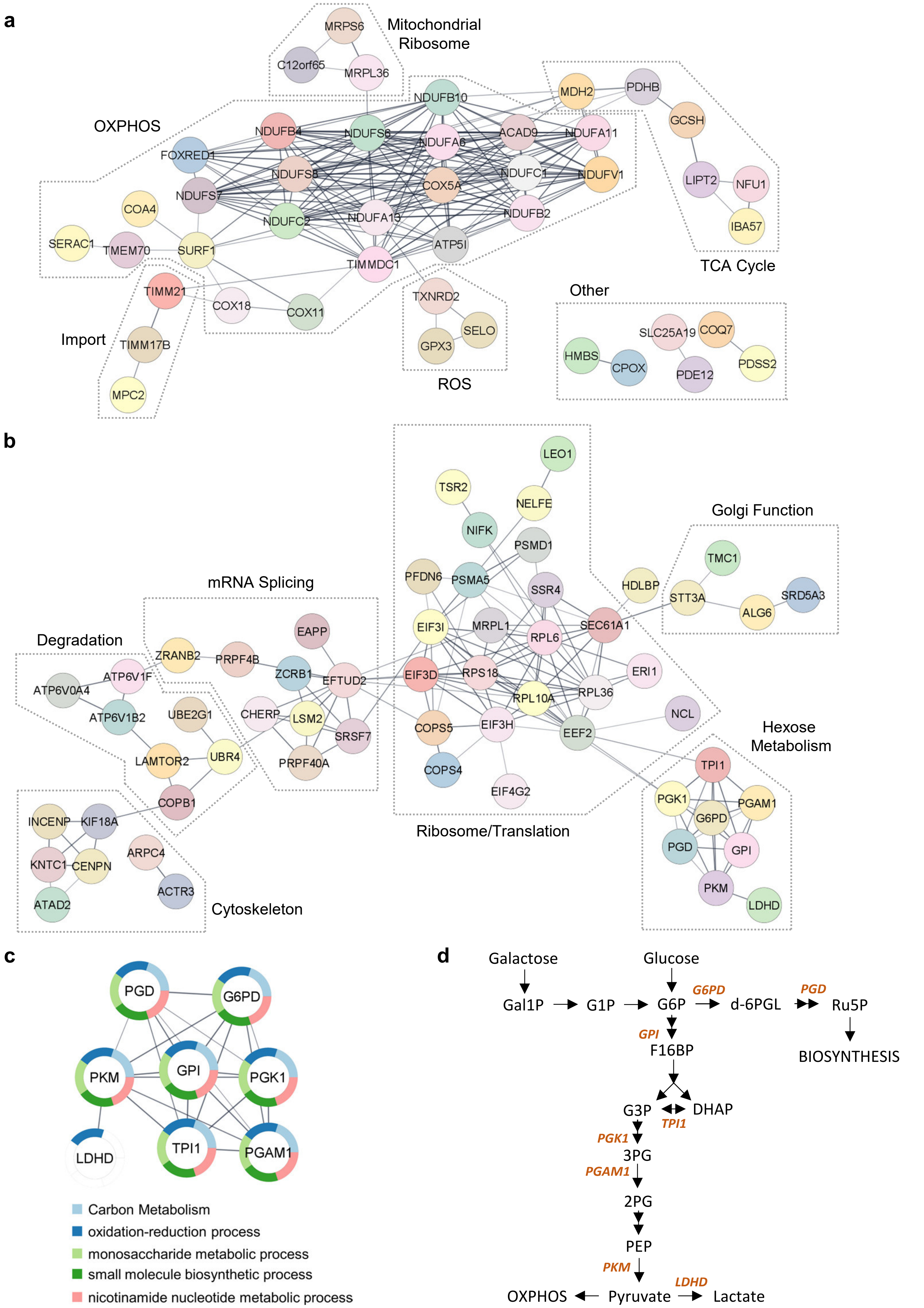

Supplementary Fig. 7: Galactose sensitises tumour cells to loss of mitochondrial genes, and promotes loss of genes involved in energy-consuming processes.

Supplementary Fig. 7: Galactose sensitises tumour cells to loss of mitochondrial genes, and promotes loss of genes involved in energy-consuming processes. a-b, Schematic diagrams show all interacting genes from genes with significantly depleted (**a**) or enriched (**b**) sgRNAs in normoxia-galactose compared to normoxia-glucose. Nodes of genes with similar functions are boxed, as indicated. **c**, Schematic diagram shows nodes of interacting genes with significantly depleted sgRNAs in normoxia-galactose involved in hexose carbon metabolism. **d**, Schematic diagram shows truncated Leloir, glycolytic and pentose phosphate pathways. Pathway genes with significantly enriched sgRNAs in normoxia-galactose compared to normoxia-glucose are highlighted in brown.

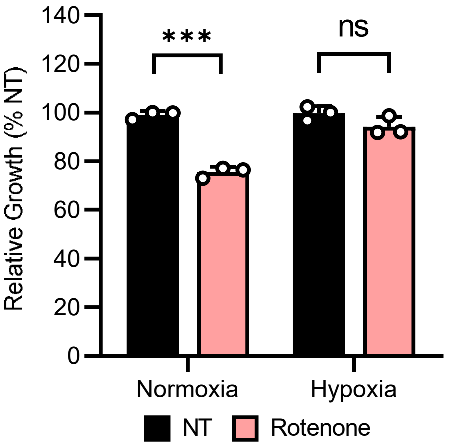

Supplementary Fig. 8: Inhibitory effects of rotenone in normoxia and hypoxia. Chart shows relative growth of U2OS cells incubated in normoxia or hypoxia (1% O_2) in glucose conditions, either untreated (NT) or treated with 31.25 nM rotenone for 72h. n=3; mean±S.D.; n.s. not significant; ***p<0.001.

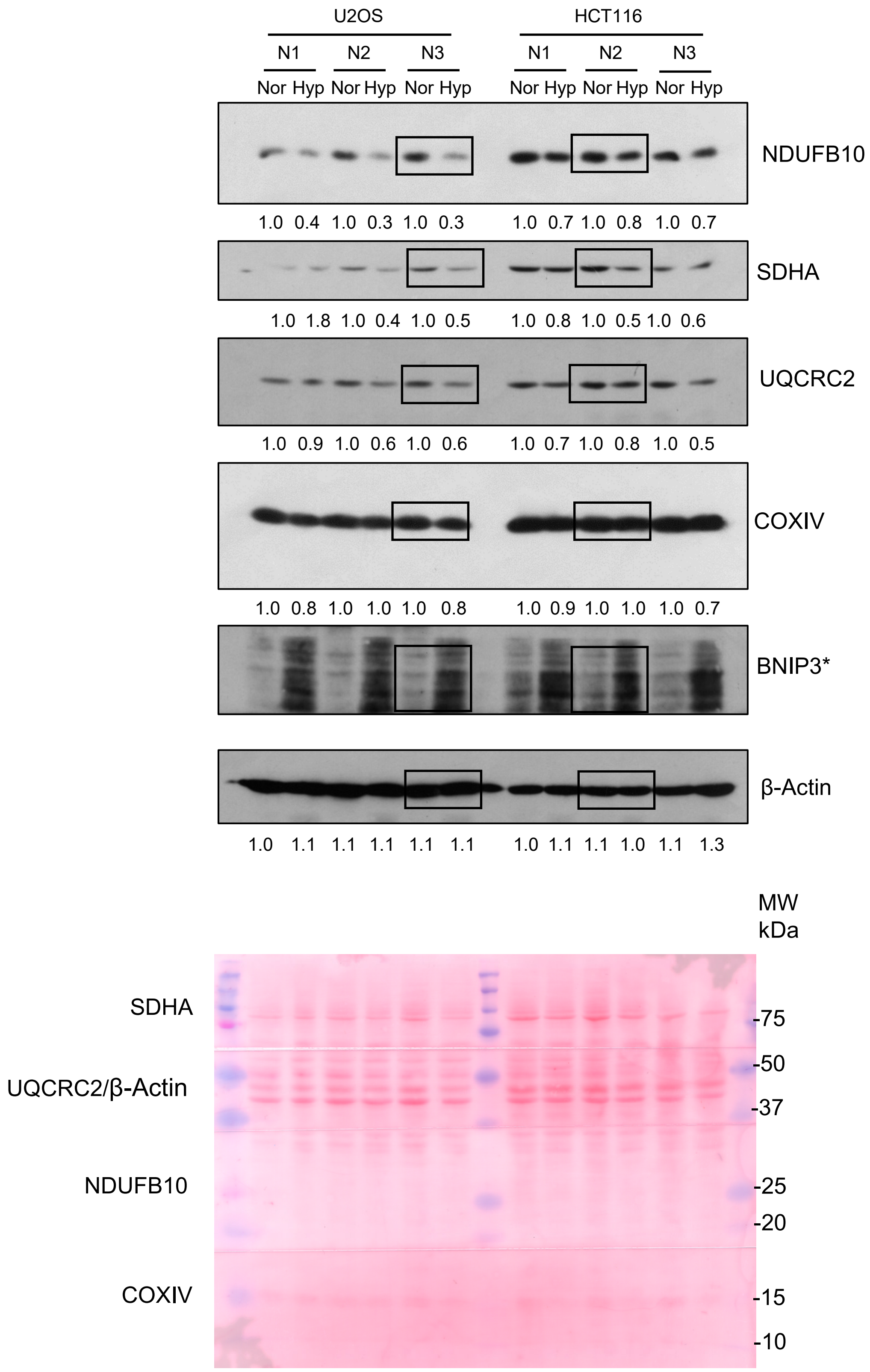

Supplementary Fig. 9: Unprocessed western blots and ponceau S-stained membranes. Related to Fig. 2e and Supplementary Fig. 4b. Densitometry value for hypoxia (Hyp) relative to 1.0 for normoxia (Nor), for each Nor/Hyp sample pair N1, N2, N3. * For BNIP3 ponceau S, see Supplementary Fig. 10.

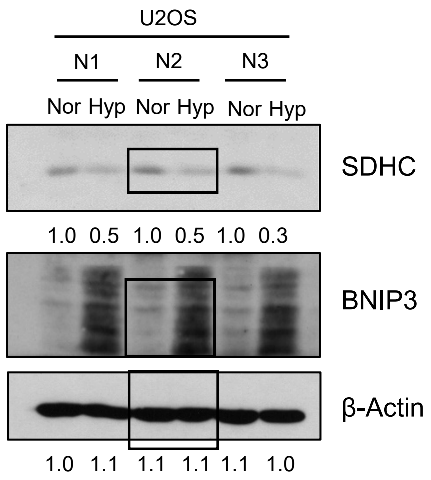

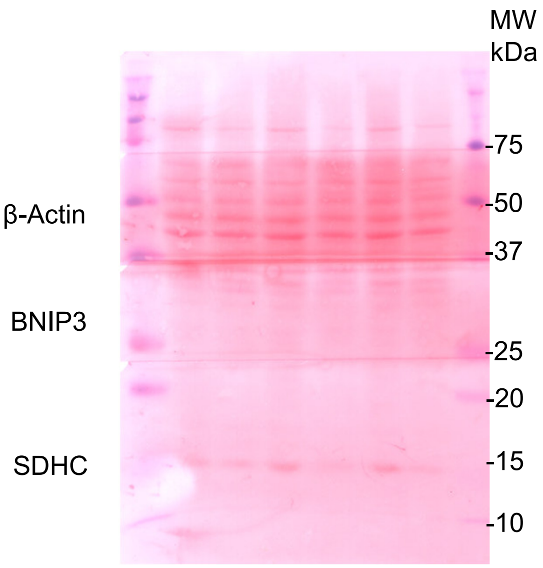

Supplementary Fig. 10: Unprocessed western blots and ponceau S-stained membranes. Related to Fig. 3c. Densitometry value for hypoxia (Hyp) relative to 1.0 for normoxia (Nor), for each Nor/Hyp sample pair N1, N2, N3.

Supplementary Information. Thomas et al.

Supplementary Tables.

Supplementary Table 1. Selected enriched mitochondrial sgRNAs for hypoxia vs normoxia. Related to Fig. 2. Compared to normoxia-glucose, we highlight 31 sgRNAs significantly enriched (FDR<30%) in hypoxia that targeted mitochondrial genes. Notably, 9 of these 31 mitochondrial genes that had significantly enriched sgRNAs in hypoxia compared to normoxia, were significantly depleted (FDR<30%) in normoxia-glucose (*).

Gene ID	Description	*p*-value
SDHC*	succinate dehydrogenase complex, subunit C, integral membrane protein, 15kDa	0.033982
PDSS2	prenyl (decaprenyl) diphosphate synthase, subunit 2	0.230311
MTERFD2	MTERF domain containing 2	0.230525
SNAP29*	synaptosomal-associated protein, 29kDa	0.280701
SOD2*	superoxide dismutase 2, mitochondrial	0.280701
POLG	polymerase (DNA directed), gamma	0.280701
MRPL35	mitochondrial ribosomal protein L35	0.280701
UQCRFS1*	ubiquinol-cytochrome c reductase, Rieske iron-sulfur polypeptide 1	0.280701
NDUFAF2	NADH dehydrogenase (ubiquinone) complex I, assembly factor 2	0.280701
PET112*	PET112 homolog (yeast)	0.280701
NUBPL	nucleotide binding protein-like	0.280701
GSTK1	glutathione S-transferase kappa 1	0.280701
DDX28*	DEAD (Asp-Glu-Ala-Asp) box polypeptide 28	0.280701
COA6	cytochrome c oxidase assembly factor 6 homolog (S. cerevisiae)	0.280701
NDUFA8	NADH dehydrogenase (ubiquinone) 1 alpha subcomplex, 8, 19kDa	0.280701
HSDL2	hydroxysteroid dehydrogenase like 2	0.280701
NDUFA12	NADH dehydrogenase (ubiquinone) 1 alpha subcomplex, 12	0.280701
ATPAF2	ATP synthase mitochondrial F1 complex assembly factor 2	0.280701
COQ7	coenzyme Q7 homolog, ubiquinone (yeast)	0.280701
OXNAD1	oxidoreductase NAD-binding domain containing 1	0.280701
REXO2	RNA exonuclease 2	0.280701
COX20*	COX20 cytochrome C oxidase assembly factor	0.280701
MRPL21*	mitochondrial ribosomal protein L21	0.280701
BDH1	3-hydroxybutyrate dehydrogenase, type 1	0.284931
POLG2	polymerase (DNA directed), gamma 2, accessory subunit	0.284931
FDX1L*	ferredoxin 1-like	0.284931
CARS2	cysteinyl-tRNA synthetase 2, mitochondrial	0.284931
GTPBP10	GTP-binding protein 10	0.284931
TWNK/C10orf2	chromosome 10 open reading frame 2	0.295728
CRIF1	growth arrest and DNA-damage-inducible, gamma interacting protein 1	0.295728
RBFA	ribosome binding factor A	0.298495

Supplementary Table 2. QPCR primer sequences. Related to Fig. 3 and Supplementary Fig. 5.

**Gene**	**Forward (5'-3')**	**Reverse (5'-3')**
SDHC	TCAAACCGTCCTCTGTCTCC	AAAGAGAGACCCCTGCACTC
EGLN3	GATGCTGAAGAAAGGGC	CTGGCAAAGAGAGTATCTG
ACTB	CCCAGAGCAAGAGAGG	GTCCAGACGCAGGATG
MTND1	GCCCCAACGTTGTAGGCCCC	AGCTAAGGTCGGGGCGGTGA
B2M	GAATGAGCGCCCGGTGTCCC	CCAAGCCAGCGACGCAGTG

Supplementary Table 3. siRNA sequences. Related to Fig. 3 and Supplementary Fig. 6.

**Gene**	**Sense (5'-3')**	**Antisense (5'-3')**
siSDHC	AAGAAGAGAUGGAGCGGUU[dT][dT]	AACCGCUCCAUCUCUUCUU[dT][dT]
