# Supplementary material for: Genome-wide CRISPR/Cas9 deletion screen defines mitochondrial gene essentiality and identifies routes for tumour cell viability in hypoxia
Source: Commun Biol. 2021 May 21;4:615. doi: 10.1038/s42003-021-02098-x (PMC8140129; doi:10.1038/s42003-021-02098-x)
Supplement: Supplementary file 3 — Description of Additional Supplementary Files [file 42003_2021_2098_MOESM3_ESM.pdf]

Description of Additional Supplementary Files.

File name: Supplementary Data.zip

Supplementary Data 1: Functional annotation of Mitocarta 2.0 genes, with common essential genes (Achilles project) highlighted. Unmodified Mitocarta 2.0, and common essential gene lists are also provided.

Supplementary Data 2: Depleted and enriched gene lists from library transduced vs plasmid U2OS-Cas9 cells, incubated in normoxia-glucose, for each of three replicates. Raw read counts (duplicate) for each of three replicates.

Supplementary Data 3: Depleted and enriched gene lists from library transduced vs plasmid U2OS-Cas9 cells, incubated in hypoxia-glucose, for each of three replicates. Raw read counts (duplicate) for each of three replicates.

Supplementary Data 4: Depleted and enriched gene lists from library transduced vs plasmid U2OS-Cas9 cells, incubated in normoxia-galactose, for each of three replicates. Raw read counts (duplicate) for each of three replicates.

Supplementary Data 5: Raw data files for charts.
