# Supplementary material for: Genome-wide CRISPR/Cas9 deletion screen defines mitochondrial gene essentiality and identifies routes for tumour cell viability in hypoxia
Source: Commun Biol. 2021 May 21;4:615. doi: 10.1038/s42003-021-02098-x (PMC8140129; doi:10.1038/s42003-021-02098-x)
Supplement: Supplementary file 5 — Reporting Summary [file 42003_2021_2098_MOESM5_ESM.pdf]

nature research

Corresponding author(s): COMMSBIO-20-1799B

Last updated by author(s): 01/04/2021

Reporting Summary

Nature Research wishes to improve the reproducibility of the work that we publish. This form provides structure for consistency and transparency in reporting. For further information on Nature Research policies, see our Editorial Policies and the Editorial Policy Checklist.

Statistics

For all statistical analyses, confirm that the following items are present in the figure legend, table legend, main text, or Methods section.

n/a	Confirmed	
☐	☒	The exact sample size (n) for each experimental group/condition, given as a discrete number and unit of measurement
☐	☒	A statement on whether measurements were taken from distinct samples or whether the same sample was measured repeatedly
☐	☒	The statistical test(s) used AND whether they are one- or two-sided<br>*Only common tests should be described solely by name; describe more complex techniques in the Methods section.*
☒	☐	A description of all covariates tested
☒	☐	A description of any assumptions or corrections, such as tests of normality and adjustment for multiple comparisons
☐	☒	A full description of the statistical parameters including central tendency (e.g. means) or other basic estimates (e.g. regression coefficient) AND variation (e.g. standard deviation) or associated estimates of uncertainty (e.g. confidence intervals)
☐	☒	For null hypothesis testing, the test statistic (e.g. F, t, r) with confidence intervals, effect sizes, degrees of freedom and P value noted<br>*Give P values as exact values whenever suitable.*
☒	☐	For Bayesian analysis, information on the choice of priors and Markov chain Monte Carlo settings
☒	☐	For hierarchical and complex designs, identification of the appropriate level for tests and full reporting of outcomes
☒	☐	Estimates of effect sizes (e.g. Cohen's d, Pearson's r), indicating how they were calculated

Our web collection on statistics for biologists contains articles on many of the points above.

Software and code

Policy information about availability of computer code

Data collection	All relevant information is noted in the text, including links to online data sources.
Data analysis	All relevant information is noted in the text, including links to online data analysis tools, and analysis software details.

For manuscripts utilizing custom algorithms or software that are central to the research but not yet described in published literature, software must be made available to editors and reviewers. We strongly encourage code deposition in a community repository (e.g. GitHub). See the Nature Research guidelines for submitting code & software for further information.

Data

Policy information about availability of data

All manuscripts must include a data availability statement. This statement should provide the following information, where applicable:

- Accession codes, unique identifiers, or web links for publicly available datasets
- A list of figures that have associated raw data
- A description of any restrictions on data availability

Genes identified from the functional annotation of all MitoCarta 2.0 genes represented in Achilles Cancer Dependency essentiality dataset described in Fig. 1, are included in Supplementary Data 1. For our CRISPR/Cas9 screens, the raw read counts, and the ranked gene lists (enriched and depleted genes) and MAGeCK analysis data (FDR-corrected p-value) for each condition and repeat screen (Supplementary Fig. 1A) are included in the Supplementary Data 2-4. The FDR-corrected significance values for all genes with significantly depleted sgRNAs at different FDR thresholds (<10%, <20%, <30%, >30%), for each condition and repeat screen are included in Supplementary Fig. 2a-c. Raw data for charts (Fig. 3b, Fig. 3d-f, Fig. 4d and Supplementary Fig. 4b) are included in Supplementary Data 5. Raw western analysis data for Fig. 2e and Fig 3c are included in Supplementary Fig. 9-10. For any further information or reasonable requests, please contact the corresponding author.

Field-specific reporting

Please select the one below that is the best fit for your research. If you are not sure, read the appropriate sections before making your selection.

☒ Life sciences ☐ Behavioural & social sciences ☐ Ecological, evolutionary & environmental sciences

For a reference copy of the document with all sections, see nature.com/documents/nr-reporting-summary-flat.pdf

Life sciences study design

All studies must disclose on these points even when the disclosure is negative.

Sample size	Each experiment has at least three biological replicates, each comprising three technical replicates (except where stated).
Data exclusions	No data were excluded.
Replication	Reproducibility was verified through biological replicates, and replication of experiments using a secondary cell line to confirm observations in the primary cell line used. Data analysis was carried out in duplicate by independent researchers to control for bias, and verify methodologies.
Randomization	N/A
Blinding	N/A

Reporting for specific materials, systems and methods

We require information from authors about some types of materials, experimental systems and methods used in many studies. Here, indicate whether each material, system or method listed is relevant to your study. If you are not sure if a list item applies to your research, read the appropriate section before selecting a response.

Materials & experimental systems

n/a	Involved in the study	
☐	☒	Antibodies
☐	☒	Eukaryotic cell lines
☒	☐	Palaeontology and archaeology
☒	☐	Animals and other organisms
☒	☐	Human research participants
☒	☐	Clinical data
☒	☐	Dual use research of concern

Methods

n/a	Involved in the study	
☒	☐	ChIP-seq
☒	☐	Flow cytometry
☒	☐	MRI-based neuroimaging

Antibodies

Antibodies used	Antibodies used were as follows, with dilutions in parentheses: rabbit monoclonal NDUFB10 (clone 13G12AF12BB11, #ab196019, 1:2000) from Abcam; rabbit monoclonal SDHA (clone D6J9M, #11998, 1:1000) from Cell Signaling; mouse monoclonal UQCRC2 (#ab14745, 1:1000) from Abcam; mouse monoclonal COXIV (clone 3E11, #4850, 1:10000) from Cell Signaling; rabbit polyclonal BNIP3 (#HPA003015, 1:2000) from Cambridge Biosciences; rabbit polyclonal SDHC (#PA5-79966, 1:1000) from Invitrogen; mouse monoclonal β-Actin (clone AC-15, #ab6276, 1:10000) antibody from Abcam; donkey anti-rabbit (#NA934, 1:1000) and anti-mouse (#NA931, 1:1000) horseradish peroxidase (HRP)-linked secondary antibodies from VWR.
Validation	NDUFB10 - Used in multiple publications. SDHA - Used in over 30 publications. UQCRC2 - Used in over 150 publications. COXIV - Used in over 200 publications. BNIP3 - highly validated 'prestige' antibody, by the manufacturers in conjunction with the Human Protein Atlas project. SDHC - validated using siRNA knockdown in-house. β-Actin - used extensively in over 1500 publications.

Eukaryotic cell lines

Policy information about cell lines

Cell line source(s)	Parental human U2OS osteosarcoma and HCT116 colon carcinoma cell lines were obtained from American Tissue Culture Collection (ATCC)
Authentication	All cell lines authenticated using STR authentication. Reports available upon request.
Mycoplasma contamination	Cell lines routinely tested, and confirmed to be negative for mycoplasma contamination.
Commonly misidentified lines (See ICLAC register)	N/A
